# Novel Bead-Based Epitope Assay is a sensitive and reliable tool for profiling epitope-specific antibody repertoire in food allergy

**DOI:** 10.1038/s41598-019-54868-7

**Published:** 2019-12-05

**Authors:** Maria Suprun, Robert Getts, Rohit Raghunathan, Galina Grishina, Marc Witmer, Gustavo Gimenez, Hugh A. Sampson, Mayte Suárez-Fariñas

**Affiliations:** 1 Department of Pediatrics, Allergy and Immunology, Icahn School of Medicine at Mount Sinai, New Yok, NY USA; 2 Department of Population Health Science and Policy, Icahn School of Medicine at Mount Sinai, New Yok, NY USA; 3 AllerGenis LLC, Hatfield, PA USA; 4 Department of Genetics and Genomic Sciences, Icahn School of Medicine at Mount Sinai, New Yok, NY USA

**Keywords:** Assay systems, Inflammatory diseases

## Abstract

Identification of allergenic IgE epitopes is instrumental for the development of novel diagnostic and prognostic methods in food allergy. In this work, we present the quantification and validation of a Bead-Based Epitope Assay (BBEA) that through multiplexing of epitopes and multiple sample processing enables completion of large experiments in a short period of time, using minimal quantities of patients’ blood. Peptides that are uniquely coupled to beads are incubated with serum or plasma samples, and after a secondary fluorophore-labeled antibody is added, the level of fluorescence is quantified with a Luminex reader. The signal is then normalized and converted to epitope-specific antibody binding values. We show that the effect of technical artifacts, i.e. well position or reading order, is minimal; and batch effects - different individual microplate runs - can be easily estimated and eliminated from the data. Epitope-specific antibody binding quantified with BBEA is highly reliable, reproducible and has greater sensitivity of epitope detection compared to peptide microarrays. IgE directed at allergenic epitopes is a sensitive biomarker of food allergy and can be used to predict allergy severity and phenotypes; and quantification of the relationship between epitope-specific IgE and IgG4 can further improve our understanding of the immune mechanisms behind allergic sensitization.

## Introduction

Food allergy is a type I immune hypersensitivity affecting 7.6% of children and 10.8% of adults in the United States^1,2^. It is characterized by the generation of allergen-specific immunoglobulin (Ig)E that binds to antigen-presenting cells and effector cells, i.e. mast cells and basophils, which following exposure to an allergen results in immediate allergic reactions^3^. The diagnosis of food allergy is determined by the oral food challenge (OFC), which arose in the 1950s when it was established that the elimination of allergenic foods resulted in a complete resolution of symptoms, while reintroduction of the allergen caused the return of those reactions^4,5^. Since then the procedure for the double-blind placebo-controlled food challenge was standardized and accepted as the “gold standard” for allergy diagnosis^6,7^. However, this procedure is burdensome for both patients and medical providers, as it takes multiple hours and carries inherent risks of severe allergic reactions. Over the years, a substantial effort has been made to refine the methods for allergy diagnosis that can complement and eventually replace OFCs.

The use of chemically extracted food fractions as a diagnostic tool for hypersensitivity dates back to 1912 when Schloss used proteins in the scratch skin test^8–10^. Different *in vivo* subcutaneous tests, i.e. Skin-Prick or Prick-Prick, are still commonly used in clinic, as they can be interpreted immediately and allow testing of multiple allergens. However, they have been shown to lack specificity^11–16^, and must be interpreted only in conjunction with carefully collected medical history.

More recent work demonstrating that *in vitro* quantification of IgE specific to allergen extracts correlated with the likelihood of allergic reactions^17–20^, fueled the development of new diagnostic assays and the search for the ideal biomarker. The ability to refine proteins that drive IgE responses, e.g. Ara h 2 in peanut^21^, gave rise to component-resolved diagnostics (CRD)^22^. Further development of diagnostic methods occurred when researchers started mapping IgE-binding epitopes, short sequences of amino acids from allergenic proteins, using SPOT membranes^23,24^. This subsequently led to a more granular understanding of the immune mechanisms of food allergy. Studies of conformational and sequential epitopes uncovered different phenotypes of allergy, e.g. children who outgrew their milk or egg allergy had IgE to predominantly conformational epitopes while those with persistent allergy possessed IgE to sequential epitopes^25,26^. As the technology continued to evolve enabling the screening of large libraries of peptides using microarrays^27^, it was found that not only IgE binding to specific epitopes, but also antibody diversity – IgE recognition of greater numbers of different epitopes - was associated with the severity of allergic symptoms^28^.

The granularity provided by the molecular-based diagnostic, i.e. peptide microarrays and SPOT membranes, gave hope of improving the accuracy of diagnostic methods. However, they came with drawbacks as SPOT membranes required large quantities of serum and did not allow clear quantification or multiple sample screening and, similarly to microarrays, required labor-intense protocols. To further advance the molecular allergy diagnostics and potentially bring it to the clinic, we have developed a Bead-Based Epitope Assay (BBEA) that allows high-throughput screening of samples and epitopes with significantly smaller volumes of serum or plasma compared to current molecular assays. BBEA provides greater sensitivity and reproducibility than microarrays and better resolution than component resolved diagnostics, making it a promising tool for the development of diagnostic and prognostic biomarkers.

Ultimately the goal of the bioassay is to detect biomarkers that have low technical variability, low baseline variation within the individual, as well as high variation between individuals and a large effect size in response to perturbation^29^. Here we present a validation of the BBEA to measure epitope-specific humoral immune profiles as potential biomarkers of food allergy. We show that allergenic epitopes detected with BBEA have high reliability within samples and reproducibility across different laboratories, large effect size across allergic and non-allergic patients, and inter-patient variability that allows discrimination between different disease phenotypes. Additionally, the high resolution provided by epitope analyses may enable further discoveries of the interplay between epitope-specific IgE and IgG4 antibodies in predicting food allergy thresholds as determined by the OFC.

## Results

### Bead-Based epitope assay

**Figure 1:**
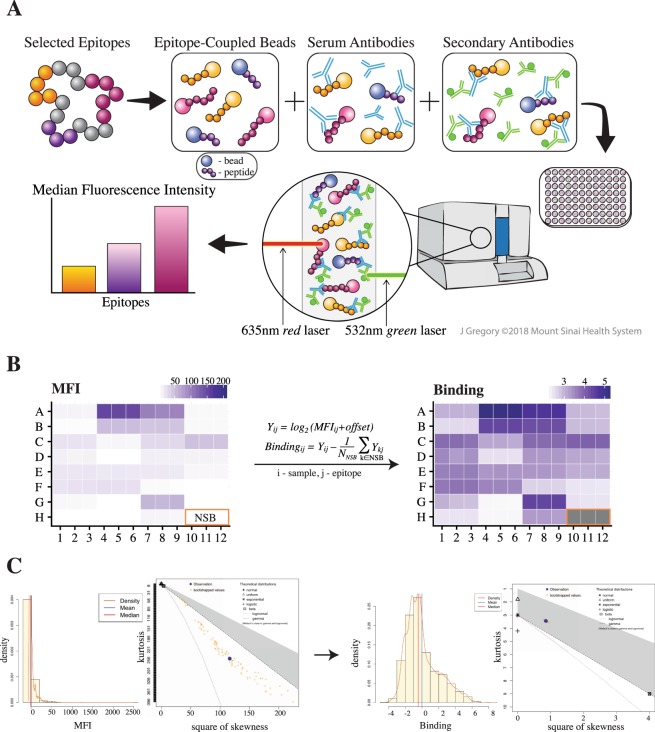
Bead-Based Epitope Assay (BBEA) set up and quantification. (**A**) Selected epitopes are uniquely coupled to beads, incubated with a patient’s serum or plasma sample and secondary antibodies. Then the 96-well plate is read on the Luminex-200 instrument, which uses dual-laser classification to detect beads and secondary antibodies, and the signal is quantified as a Median Fluorescence Intensity (MFI). *Illustration by Jill Gregory. Printed with permission from Mount Sinai Health System, licensed under CC BY-ND*. (**B**) Image of the 96-well plate with MFI is then transformed to binding scores using log_2_ normalization and subtraction of the non-specific binding (NSB) wells. (**C**) Histograms and Cullen-Frey plots of the MFIs and binding scores indicate that MFI distribution has large skewness and kurtosis, while binding scores are closer to normal or log-normal distributions.

The bead-based epitope assay (BBEA) enables simultaneous quantification of antibodies binding to different sequential epitopes for multiple samples. Epitopes are first covalently coupled to unique fluorescent microspheres (Luminex beads). The epitope-loaded beads are then mixed together to form a multiplex master library and added to a 96-well microplate (Fig. 1A). To identify epitopes that are bound by patients’ antibodies, the master library is first incubated with a patient’s serum or plasma, and then with the secondary fluorophore-labeled antibody. To detect and later eliminate secondary antibodies that directly recognize peptides or beads, i.e. non-specific binding (NSB), wells with only the master epitope library and secondary antibodies are included (Fig. 1B). The plates are read with a Luminex instrument that uses dual-laser classification, where the red laser detects beads and the green laser identifies secondary antibodies; and for every epitope the signal is quantified as a median fluorescence intensity (MFI).

The BBEA doesn’t limit epitope detection to any specific antigen and allows researchers to customize peptide libraries and antibodies. In this study we present a quantification characterization of IgE and IgG4 binding to milk and peanut epitopes using BBEA. The milk and peanut libraries were designed and created based on published experiments^28,30–38^ and consisted of 66 epitopes from the 5 most clinically relevant milk proteins, i.e. αS1-casein, αS2-casein, β-casein, β-lactoglobulin, and κ-casein; and 50 epitopes from the 3 major peanut allergens, i.e. Ara h 1, Ara h 2, and Ara h 3.

Initial quantification of MFI and bead counts underwent quality control and normalization. Bjornstal *et al*.^39^ previously showed that using at least 25 beads per antigen/peptide produces consistent MFIs, while using lower bead counts resulted in increased variability between identical samples. Samples with an average bead count less than 30 were thus excluded from the downstream analyses. The Cullen-Frey analysis^40,41^, which shows the parameters of theoretical distributions across 100 bootstrap simulations, indicated that the original distribution of the MFIs was generally skewed (Fig. 1B,C). The MFI was then normalized and converted to binding scores using log_2_ transformation with an added 0.5 constant, and a subtraction of the average of NSB wells (Fig. 1B). These binding scores had a skewness and kurtosis closer to those of the normal and/or log-normal distributions (Fig. 1B,C), allowing further characterization of the assay using parametric modeling.

### Identification and elimination of technical artifacts

#### Well effect

**Figure 2:**
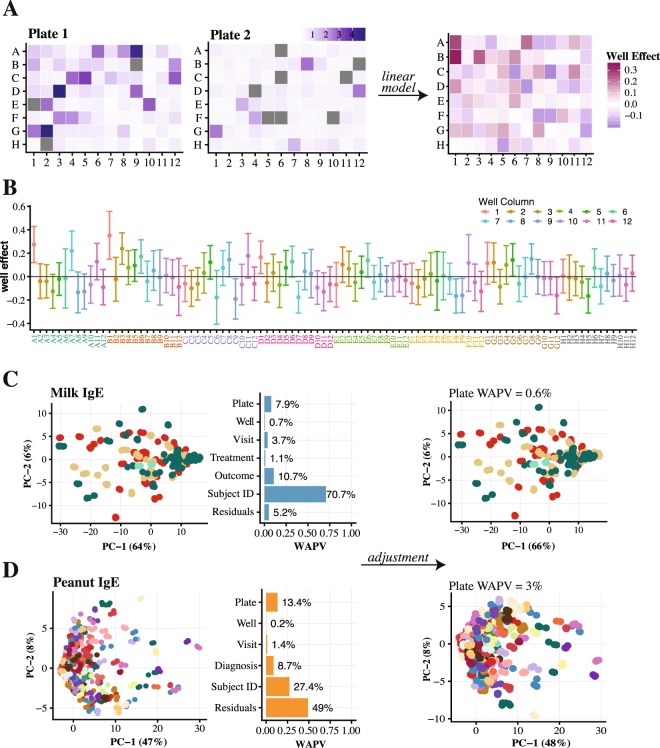
Detection and elimination of technical artifacts. (**A**) Images of the 2 plates with a randomized position of technical triplicates, and, on the right, the image of the coefficients for each well estimated with the linear mixed-effects model of those two plates (a coefficient of zero is indicative of no effect). (**B**) Estimated coefficients with 95% CIs for each well, presented in the order they were read by Luminex-200. With the majority of the coefficients centered around zero, no systematic bias attributable to the well position is observed, with slightly higher effect of the A1 and B1 wells. (**C**,**D**) On the left, PCA and PVCA plots for the 4 milk and 22 peanut IgE plates, showing that 8% and 13% (quantified as the weighted average proportion variance, WAPV) of the variability in the data is attributable to the plate effect. PCA plots on the right show that after the adjustment, this effect is responsible for <1% and <3%, for milk and peanut respectively. On the PCA plots points represent individual samples done in triplicates colored by the plates (batches).

Microplate experiments are known to have well position and edge effects, where wells on the edges of the plate tend to give different results due to more rapid liquid evaporation^42–45^. Another potential source of bias is the well reading order, which starts at A1 to A12, then B1 to B12, until well H12 is reached. This leaves lower rows (F-H) incubating for a longer period of time (>1 hour) and consequently differences might be expected to occur between earlier and later rows. To investigate whether this is the case for BBEA, two plates of a peanut experiment had a completely randomized design (Fig. 2A), with triplicates randomly positioned within plates. Using these data, the well effect was estimated using a mixed-effects model, and no systematic noise was observed in the data that could be attributed to either rows, columns, or the reading order of the microplate (Fig. 2A). Overall, the effect of A1 and B1 seemed to be higher than the rest of the wells (Fig. 2B), but this was likely due to a small sample size rather than true position effect, which should cause lower binding across the edge wells. After observing no position effect of the triplicates, the rest of the experiments were done with the replicates located in adjacent wells, which makes the dispensing of the samples faster, reducing the time of assay preparation and possibility of procedural errors.

#### Plate effect

High-throughput assays have well documented batch effects, where each experiment carries specific technical artifacts that can confound biological signals^46–48^. In the BBEA, the batch is considered an individual microplate run, which can be influenced by a variety of factors, e.g. different technicians, room temperatures or lighting. We have investigated the presence of plate effect using principal component analysis (PCA) and principal variance component analysis (PVCA) in milk and peanut experiments^49^.

In the milk study, plate effect was seen on the PCA plots and estimated by the PVCA to account for 8% and 2% of total variability, for IgE (Fig. 2C) and IgG4 (Fig. S1A) (For clarity IgE figures are presented in the manuscript while IgG4 are included in supplementary materials). This effect was easily eliminated using a multivariable linear model where the plate coefficients were estimated for each peptide along with experiment-specific factors, in this case visit and treatment, and then subtracted from the original binding values. In the adjusted data this batch effect was eliminated for both IgE and IgG4. Similar to the milk study, analysis of peanut experiments revealed plate effects for both IgE and IgG4, accounting for 13% and 31% of variability (Figs. 2D and Fig. S1B). Again, the plate effect was easily adjusted for by linear modeling, as a result accounting for only 3% in IgE and 0% in IgG4 experiments. The model fitting was necessary for every peptide since we have observed that peptides with higher binding showed more variability and carried higher estimated plate effect in both IgE and IgG4 peanut experiments (Fig. S2).

The plate estimation was quite straightforward since the experiment was designed ensuring random allocation of disease phenotypes and treatment groups across plates. As such, there was no confounding between experimental conditions and plates, and both factors could be adequately estimated. The milk study was relatively small and included only 4 plates for each antibody, which made it possible to empirically randomize the plates. However, for larger experiments, such ad hoc randomization may not always prove successful. As an added quality assurance measure, we have developed *PlateDesigner*^50^, a web application specifically for the sample randomization across microplates. This design software may be applied to any study and was used for the design of the CoFAR2 peanut experiment.

### Reliability and reproducibility of BBEA

An important characteristic of any assay is that it is both reliable and reproducible. Reliability refers to the agreement between multiple measures assessed on the same sample under the same conditions; while reproducibility looks at the variation of the measurement on the same sample under varying conditions, i.e. different instruments or observers. We have assessed the reliability and reproducibility of the BBEA with a separate set of experiments where IgE and IgG4 binding to peanut epitopes was measured by 3 independent laboratories using Luminex-200 instruments.

**Figure 3:**
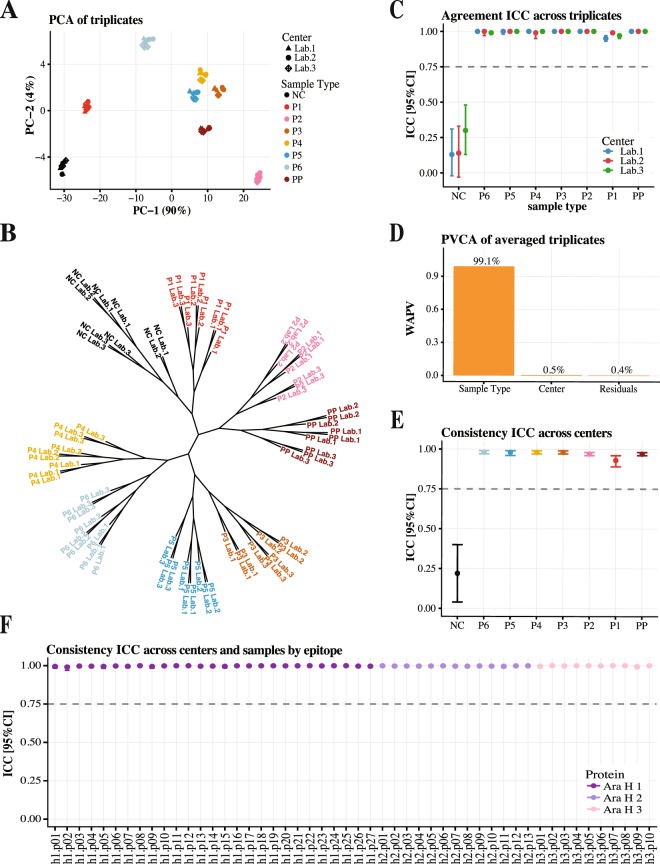
Reliability and reproducibility of the IgE binding to peanut epitopes. (**A**) PCA plot of the technical triplicates colored by the sample type. (**B**) Phylogenetic tree of the technical triplicates constructed with the Spearman correlation as distance metric and “average” agglomeration algorithm. (**C**) Average agreement ICC with 95% CI across triplicates for all samples within three centers. These results indicate high reliability of the BBEA with triplicates from the same samples clustering together and having high agreement; with the exception of the NC that has MFI and binding scores close to 0. (**D**) PVCA plot shows that the center contributes to a small proportion of total variance (WAPV <1%), with the majority of variance explained by the sample type. (**E**) Average consistency ICC with 95% CI across the 3 centers for all 8 samples. (**F**) Average consistency ICC with 95% CI across the 3 centers for all 50 epitopes. These results show that BBEA has high reproducibility across independent laboratories.

Utilizing a standardized set of samples and procedures in each laboratory, the reliability across technical triplicates was assessed for 8 samples that represented pools of patients with varying degrees of clinical peanut reactivity, including a non-allergic control patient (NC). PCA plots and phylogenetic trees showed evident clustering of triplicates belonging to the same samples (Figs. 3A,B and S3AB). Overall, the reliability across replicates was “excellent” according to the guidelines^51^, with the intra-class correlation coefficient (ICC) for agreement >0.9 for IgE (Fig. 3C) and >0.75 for IgG4 (Fig. S3C). Of note, as NC is not expected to have IgE to the allergenic epitopes, their MFI and binding scores were close to zero (mean 0.09) much smaller than the standard deviation (SD = 0.28), resulting in low agreement (ICC = 0.19) for IgE. The confidence intervals of the agreement ICC for IgG4 (Fig. S3C) had higher variability compared to the IgE results (Fig. 3C). This was driven by a small additive shift in one of the triplicates, even though the replicates were highly correlated and had high ICC for consistency (Table S1).

BBEA’s reproducibility across the three laboratories was evaluated using the average binding of the technical triplicates. PVCA analyses showed much more of the variance in the data attributed to the sample type than to the laboratory, with a weighted average proportion variance (WAPV) of 99% versus 0.5% for IgE (Fig. 3D) and 67% versus 30% for IgG4 (Fig. S3D). Consistency ICC was also high for both IgE (Fig. 3E) and IgG4 (Fig. S3E) across peanut-reactive samples, and for all 50 epitopes (ICCs > 0.98 for IgE; Fig. 3F and >0.8 for IgG4; Fig. S3F). We have demonstrated earlier that potential batch effect can be identified and adjusted for in BBEA (Figs. 2 and Fig. S1) and as such we have presented the ICC for consistency (which evaluate agreement minus an additive constant). If one were to proceed with the analysis of the data combined across centers, the adjustment for the batch (laboratory) effect would be a necessary initial step. After the adjustment, the clustering of triplicates for IgG4 is by samples type (Fig. S3G), and the average ICC for agreement was 0.998 for IgE and 0.94 [0.92–0.95] for IgG4.

### Benchmarking BBEA against peptide microarray

The current method to study allergenic epitopes with microarray immunoassays (MIA) allows screening of large libraries of peptides simultaneously^27,52^. However, MIA involves complex and lengthy protocols, and takes ~2 days from the start of the experiment to acquisition of the raw data. The goal of BBEA is to simplify and streamline the detection of epitope-specific antibody binding, thus shortening the procedure time while also increasing the sensitivity of the signal detection.

**Figure 4:**
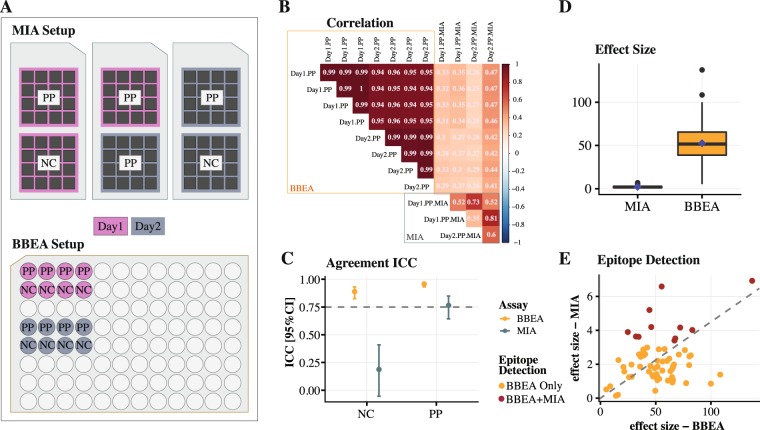
Benchmarking of BBEA against MIA using milk epitope-specific IgE. (**A**) Design of the experiment: for both BBEA and MIA same experiment was repeated on two separate days, as represented by pink and grey background colors. Each MIA experiment included two chips for the same PP and one for the NC. Each BBEA run had 4 technical replicates of both PP and NC. (**B**) Spearman correlation between replicates with higher color intensity representing stronger correlation (red - positive, blue - negative; all correlations were significant, p < 0.05). On average, correlation between replicates is close to 1.0 for the BBEA and around 0.6 for MIA. (**C**) Average agreement ICC with 95% CI across the two days is higher for BBEA (orange color) compared to MIA (grey color). (**D**) Boxplot of the effect sizes shows that BBEA can detect smaller difference. (**F**) Scatter plot of the effect sizes show that BBEA detected 66/66 milk epitopes compared to 11/66 detected by MIA. Epitope detection threshold was set to p < 0.05.

To benchmark the performance of the BBEA to MIA, we’ve performed a separate set of experiments using milk peptides. For the MIA experiment, a positive pool (PP) and NC samples were run on two separate days, and the PP was done in duplicates. For the BBEA experiment, the PP and NC were repeated in replicates of 4 on two separate days (Fig. 4A). While MIA was performed for the whole set of 235 peptides, only the subset of 66 peptides that were part of the milk epitope library were included in the analysis. Agreement among replicates was much higher for the BBEA compared to MIA (Fig. 4B), with an average correlation of 0.97 (SD = 0.02) and 0.59 (SD = 0.16), respectively. After averaging the replicates across the two days, the agreement for BBEA was high for both the PP and NC with ICCs of 0.96 with 95% CI [0.93–0.97] and 0.89 [0.83–0.93], and only moderate to low for MIA’s PP and NC with ICCs of 0.76 [0.64–0.85] and 0.19 [−0.05–0.41], indicating BBEA has greater reproducibility compared to MIA (Fig. 4C).

We then compared the ability of BBEA and MIA to identify epitopes that differ between PP and NC. 100% (66/66) of the epitopes were detected by BBEA (detection threshold p < 0.05) and only 17% (11/66) by MIA, indicating that BBEA had superior sensitivity (Fig. 4E). The effect size of BBEA was also much higher compared to MIA with a positive correlation among the two assays (r = 0.29, p = 0.028; Fig. 4D,E). Since detection based on p-values is dependent on sample size, which is larger for BBEA than MIA, we ran a sensitivity analysis randomly selecting a subset of BBEA samples to match the sample size of MIA. The results were almost identical in terms of the effect size and peptide detection rate of 98%. Furthermore, to guarantee that the conclusions of this comparison were not due to a more or less pervasive batch effect in each technology, all analyses were repeated for the batch adjusted data, where the plate effect, accounting for 3.1% of variability in BBEA and 8% in MIA, was removed. The results for the adjusted data were almost identical (Fig. S4) to the unadjusted analysis with slightly higher epitope detection rate of 21% (14/66) for MIA.

### Epitope-specific antibody binding as a biomarker of food allergy

In IgE-mediated food allergy, protein recognition by IgE antibody is indicative of allergic sensitization; and it has been previously demonstrated that epitope-specific IgE binding is associated with different disease phenotypes^28,33,36,38,53^. IgG4 is considered a blocking antibody, as it could potentially prevent allergic symptoms by out-competing IgE on effector cells for allergen. While the measurement of IgE and IgG4 to component proteins of an allergen, by microarray assays and ImmunoCAP, provides insights into the mechanism of food allergy, there are still no universally accepted set thresholds for their diagnostic utility. We sought to further investigate the relationship between IgE and IgG4 directed at allergenic epitopes in association with milk allergy severity and peanut allergy diagnosis.

**Figure 5:**
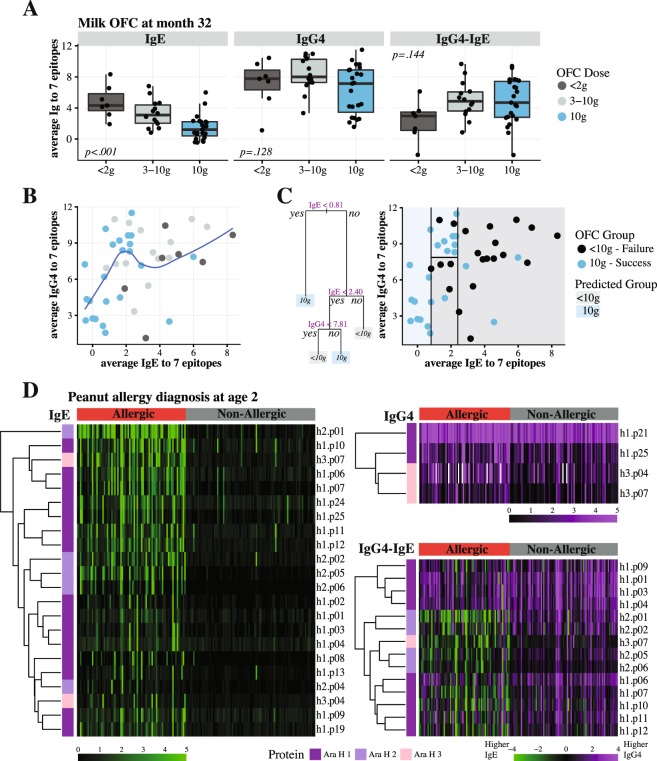
Relationship between epitope-specific IgE and IgG4 antibodies. (**A**) Boxplots of the average of 7 milk epitopes show dose-depended relationship between IgE and the OFC dose at month 32 of 44 patients; while the relationship is less clear for either IgG4 or IgG4-IgE ratio. (**B**) Scatterplot with the loess smoothed line of the average IgE and IgG4 to 7 milk epitopes; each point represents an individual patient, colored by the amount of protein they were able to consume during the OFC at month 32. (**C**) A classification tree determining best relationship between IgE and IgG4 and the binary outcome of the OFC, shows that having very high or low levels of IgE is enough to classify the OFC outcome, while at the intermediate IgE levels (0.81–2.4), high IgG4 (>7.81) can further improve classification. (**D**) Heatmaps of 73 peanut allergic (red) and 83 non-allergic (grey) patients and their IgE, IgG4, and IgG4-IgE binding to epitopes from 3 peanut proteins. The epitopes with FCH≥1.5 and FDR <0.05 are presented. All IgG4-IgE epitopes are also present among the IgE ones, indicating that IgE binding alone is most informative in identifying differences among allergic and non-allergic individuals.

Using a cohort of 47 milk allergic patients (7–35 years old) that underwent milk oral immunotherapy (OIT) ± omalizumab in a prospective placebo-controlled randomized clinical trial^54,55^, we measured IgE and IgG4 binding to 66 milk epitopes. After OIT was discontinued for 8 weeks, patients underwent final OFC with 10 g of milk protein at month 32 of the trial. Epitope-specific IgE profiles were highly correlated with IgE specific to milk component proteins and with the OFC outcome. The overall z-score for all 66 IgE epitopes had a Pearson’s correlation of 0.81 for milk, 0.87 for casein, and 0.78 for beta-lactoglobulin (p < 0.01 for all), while for individual epitopes these correlations ranged from 0.34 to 0.85. On the other hand, IgE binding to 7 epitopes most correlated with OFC, showed a dose-dependent relationship with the OFC outcome (p < 0.001), while IgG4 (p = 0.128) or the ratio of the two antibodies (IgG4-IgE, p = 0.144) had no clear pattern (Fig. 5A). We observed that while IgE and IgG4 values were positively correlated (Spearman rho = 0.43, p = 0.004), the relationship was not necessarily linear (Fig. 5B). A simple classification tree with 4 nodes correctly classified OFC outcome in 38/44 subjects (86%, AUC = 0.87) suggesting that, regardless of IgG4, having low IgE values (<0.81) would result in a successful OFC, while high IgE (>2.4) was indicative of OFC failure. However, subjects with intermediate IgE and high IgG4 (>7.81) were more likely to pass OFC as compared to those with low IgG4 (Fig. 5C).

We have considered whether IgE and IgG4 specific to peanut epitopes can better discriminate between patients who are sensitized compared to those who are clinically reactive to peanut. One hundred and sixty blood plasma samples from 2-year-old children (73 allergic and 83 non-allergic) recruited for the NIH-sponsored CoFAR2 natural history cohort^56,57^ were analyzed. Allergic diagnosis was based on either OFC, or IgE to peanut ≥14kU_A_/L, or IgE to peanut ≥0.35kU_A_/L and Skin-Prick test ≥3 mm plus a suggestive history. IgE specific to 22 epitopes had significant binding differences (fold change (FCH)≥1.5 and FDR <0.05), while only 4 IgG4 epitopes and 14 IgG4-IgE epitopes showed significant differences (Fig. 5D). All the IgG4-IgE epitopes were a subset of IgE epitopes and as shown on the heatmaps, they present a mirror image of the IgE binding rather than contributing additional information. These results indicate the IgE binding to peanut epitopes and not IgG4 or their ratio is most informative for making a diagnosis of food allergy.

## Discussion

We have developed a Bead-Based Epitope Assay to measure antibody responses directed at allergenic epitopes. The assay enables multiplexing of epitopes, allowing multiple results from a single specimen, and is done in a 96-well plate, streamlining multiple sample processing within one experiment (Fig. 1A). The BBEA protocol and acquisition of raw data for a full plate can be obtained in 4–5 hours and requires about 10 μl of patient sample per well and 60–73 ng of a single peptide for a 96-well plate. The assay output is a Median Fluorescence Intensity that is then normalized and transformed to binding scores (Fig. 1B,C) that follow normal or log-normal distributions.

High-throughput assays are known to have technical artifacts that may confound biological signals. We have considered both well and plate effects as potential sources of bias and showed that neither well position nor reading order influenced binding values (Fig. 2A,B). This allows the placement of triplicate samples in adjacent wells rather than the need to utilize a completely randomized sample distribution. After the samples are added to the plates, identifiers have to be entered into the microplate reader’s software, specifying sample to well mapping; and keeping the triplicates together allows for a less time-consuming plate preparation and reduces the potential for procedural and data mapping errors.

We have identified a batch effect that is indicative of individual microplate runs, and as shown in Figs. 2 and Fig. S1, this plate effect was easily estimated and eliminated from the data. Currently there are many ways to quantify and account for batch effects, i.e. linear modeling^58,59^ or ComBat^60^; but in order for them to work, there should be no confounding between the batch and experimental variables. For example, if samples from all allergic patients are processed on one plate and non-allergic individuals on another, it is not possible to reliably estimate how much of the result is attributable to either plate effect or allergy diagnosis alone. To avoid such scenarios, samples must be randomized across and within plates. To achieve this, large experiments with peanut epitopes were designed with *PlateDesigner*^50^ and the original plate effect that accounted for > 10% of the variability was successfully eliminated.

Epitope-specific antibody binding detected by BBEA is reliable and reproducible (Figs. 3 and Fig. S3), with high agreement among triplicates of the same samples; which could allow a reduction in the number of technical replications, especially for the IgE, and further minimize the amount of patient’s sample required for the assay. When the same experiment was repeated across three independent laboratories, consistency of the results for both samples and peptides remained excellent.

Biomarkers for allergic sensitization include allergen recognition by IgE antibodies, which are the least abundant isotype in serum, making it the most challenging to detect. We showed that the BBEA provides greater sensitivity for detection of allergenic epitopes compared to the microarray immunoassay (Fig. 4), capable of detecting larger effect size as well as larger numbers of IgE epitopes. In addition, the BBEA protocol and data acquisition are more rapid compared to MIA.

Sackesen *et al*.^61^ have shown using BBEA that antibody binding to milk epitopes discriminates between different phenotypes of milk allergy, with highest epitope-specific IgE in children reactive to baked-milk, followed by fermented milk, and then whole milk. Suarez-Farinas *et al*.^55^ developed prognostic models that were able to identify subjects that would achieve sustained unresponsiveness (remission) to milk OIT. The best model consisted of IgE epitopes and had better performance than the epitope-specific IgG4, CRDs, or combinations of those biomarkers. IgG4 antibody is considered to play a protective (blocking) role in allergy, as it competes with IgE for allergen binding^16,62–68^, and a higher ratio of IgG4 to IgE could be indicative of better outcomes and less severe allergic phenotypes^69–74^. Some groups have found either no significant association between IgG4 to IgE ratios of milk proteins and allergic phenotypes^36^, or possible effect modification by IgG4 rather than a linear relationship^75,76^. Using IgE and IgG4 binding profiles to milk epitopes, we showed that the relationship between the two antibodies is not necessarily linear (Fig. 5B) and considering the ratio diminished the association between IgE and allergy severity as defined by the OFC dose (Fig. 5A). Having low or high IgE to allergenic epitopes determined if a patient would be able to pass an OFC to 10 g of milk protein; however, at the intermediate IgE levels, also considering IgG4 resulted in improved estimations, with high IgG4 indicative of better outcomes (Fig. 5C). We then analyzed the relationship between IgE and IgG4 binding to 50 peanut epitopes in a cohort of 160 children^56^. We showed that epitope-specific IgE alone was sufficient to distinguish between peanut allergic and non-allergic children; with IgG4 and IgG4-IgE ratio providing no additional information (Fig. 5D). Larger studies of epitope-specific antibody responses might be helpful in determining the role of IgG4 and different levels of peanut reactivity.

BBEA can be extended to include a large number of peptides, only limited by the instrument capacity. The machine used, Luminex-200, can simultaneously read up to 100 different bead regions; however, other instruments, i.e. Luminex FLEXMAP 3D, can multiplex up to 500 microspheres per sample. One limitation of the assay is that the plate reader can only measure one antibody isotype at a time, and if multiple immunoglobulins need to be measured, a separate experiment must be run for each isotype. However, the procedure to measure antibodies only differs in the type and amount of the secondary antibody added and is easily extended to study multiple antibody types as unique biomarkers.

We have demonstrated that BBEA is a sensitive and reliable tool for the detection of antibodies specific to sequential allergenic epitopes, and epitopes have the potential to serve as biomarkers of food allergy. We have developed the quality control and quantification pipeline and are planning to release an R package specifically to evaluate this type of data. BBEA allows rapid quantification of allergenic epitopes in a large number of individuals, providing a great tool for identification of population differences or disease dynamics over time, and helps the discovery of novel immune mechanisms of allergy.

## Materials and Methods

### Bead-Based epitope assay protocol and signal processing

C-terminal biotinylated peptides (CS Bio, Menlo Park, CA, USA) were conjugated to the LumAvidin xMAP microspheres (Luminex Corporation, Austin, TX, USA) and stored in PBS-TBN buffer (1xPBS with 0.02% Tween-20 and 0.1% bovine serum albumin). The peptide-coupled beads are stable for up to 1 year at 4–8 C, when prepared with 0.05% Sodium Azide in the storage buffer. A master mix of microspheres was then made in PBS-TBN buffer and 100 μL/well of mix was added to 96-well filter plates. After washing the microspheres with PBS-TBN, 100 μL of plasma samples diluted 1:50 for milk- and 1:10 for peanut-allergic patients was added to the wells in triplicates. Plates were incubated on a shaker for 2 hours at 300 rpm at room temperature. Excess plasma was then removed and the plate was washed. 50 μL/well of R-phycoerythrin (PE) labeled mouse anti-human IgE-PE (2 μg/mL, Cat. No MA1-10375, Thermo-Fisher Scientific) or IgG4-PE (0.25 μg/mL, Cat. No 9200-09, Southern Biotech) secondary antibody was added and plates were incubated for 30 minutes at room temperature. After a final wash, 100 μL of PBS-TBN was added to each well to re-suspend the microspheres, which were then transferred to fixed-bottom 96-well reading plates and quantified with the xPONENT® software on Luminex200 instrument (Luminex Corporation, Austin, TX, USA).

Median Fluorescence Intensity (MFI) was obtained directly from the Luminex xPONENT® software. For the non-specific signal detection for every peptide, each plate included 3 wells with only PBS-TBN buffer and no plasma sample. For each sample i, and epitope j, the binding measurement B_ij_ was defined as:

$${Y}_{ij}=lo{g}_{2}(MF{I}_{ij}+0.5)$$

$${B}_{ij}=\{\begin{array}{cc}0, & {Y}_{ij} < \frac{{\sum }_{k{\epsilon }NSB}{Y}_{kj}}{{N}_{NSB}},\\ {Y}_{ij}-\frac{{\sum }_{k{\epsilon }NSB}{Y}_{kj}}{{N}_{NSB}}, & otherwise\end{array}$$

where *NSB* represents non-specific binding (i.e., PBS-TBN buffer) wells. This quantitative outcome denotes epitope-specific antibody binding.

### Peptide libraries

The libraries of 20-mer milk and 15-mer peanut peptides (CS Bio, Menlo Park, CA, USA) were designed based on the meta-analyses of BBEA and previously published SPOTs membrane and peptide microarray studies^28,30–38,77^. The milk library consisted of 66 peptides from αS1-casein (n = 18), αS2-casein (n = 13), β-casein (n = 14), β-lactoglobulin (n = 8) and κ-casein (n = 13) proteins. The peanut library had 50 peptides from Ara h 1 (n = 27), Ara h 2 (n=13), and Ara h 3 (n=10).

### Experimental set-up

The validation of the assay was done using several experiments: (i) milk OIT^54,55^ and (ii) peanut CoFAR2^56^ cohorts for the quantification and detection of technical artifacts, as well as the analysis of IgE and IgG4 in relation to allergy severity and diagnosis, (iii) pooled samples for the reliability and reproducibility study, and (iv) an allergic pool and a negative control for the comparison of BBEA and MIA. The assay for milk OIT cohort used 4 IgE and 4 IgG4 plates; while peanut CoFAR2 cohort was run on 22 IgE and 28 IgG4 plates. The first 2 IgE plates had all triplicates randomly assigned to wells, in order to stimate an effect of well position. After ascertaining the presence of a plate effect in milk OIT, the intraplate controls (IPCs, a pool of experimental samples) were included in triplicates on all plates of the CoFAR2 experiment. The reliability and reproducibility experiment consisted of 8 different samples, each was a pool of patients with different peanut reactivity, and measured IgE and IgG4 to peanut epitopes. This experiment was repeated by 3 independent laboratories, located in the Icahn School of Medicine at Mount Sinai (New York City, NY), AllerGenis (Hatfield, PA), and Carrera Bioscience (Ithaca, NY). With the exception of the MIA study, all samples were processed in technical triplicates. BBEA versus MIA experiment measured IgE to milk epitopes and consisted of the replication of positive (milk allergic) pool and one negative control, and was repeated on two separate days (Fig. 4A).

CoFAR2 and milk OIT studies were funded by the NIH, AI-66738 and AI-44236. Milk OIT + omalizumab clinical trial was registered on the ClinicalTrials.gov, NCT01157117. All protocols and consent/assent forms were reviewed and approved by the Program for the Protection of Human Subjects (PPHS) at the Icahn School of Medicine at Mount Sinai and the Internal Review Boards of participating institutions: milk OIT – Johns Hopkins University; CoFAR2 – Johns Hopkins University, Arkansas Children’s Hospital, University of North Carolina and National Jewish Health. Informed consents/assents were obtained for all subjects. All research methods were performed in accordance with relevant guidelines and regulations.

### Peptide microarray immunoassay

Six plasma samples from milk allergic patients were mixed 1:1 to generate a positive control pool (PP); and a plasma sample from one non-allergic participant was used as the negative control (NC). MIA was performed as previously described with some modifications^34,36,37^. In brief, a library of 235 peptides consisting of 20 amino acids overlapping by 17 (3 offset), corresponding to the primary sequences of αS1-, αS2-, β-, and κ-caseins and β-lactoglobulin was printed in 2 sets of triplicates (6 spots per peptide) onto Arrayit SuperEpoxy glass slides (Arrayit Corporation, Sunnyvale, CA, USA). The slides were blocked with 400 μL of 1% human serum albumin (HSA) in PBS containing 0.05% Tween 20 (PBS-T) for 1 hour at room temperature, then incubated with 250 μL of patient serum diluted 1:5 in PBS-T/HSA at 4 °C overnight. Slides were then washed with PBS-T and incubated for 1 hour at room temperature with a mixture of 3 monoclonal mouse anti-human IgE and IgG4 antibodies (working concentration, 0.4 μg/mL; mouse anti-human IgE from BD Biosciences Cat 555858, clone G726 and Invitrogen Cat 05-4740, and biotinylated mouse anti-human IgG4 from Southern Biotech Cat 9200-02)^36^. Slides were then incubated for 3 hours at 31 °C with a cocktail of Anti-BiotinDendrimerOyster 550 (350) and Anti-fluorescein isothiocyanate Dendrimer Oyster 650 (350) in Dendrimer buffer (Genisphere, Hatfield, PA, USA), both at 0.6 μg/mL with the addition of 0.02 μg/mL of salmon sperm DNA (Invitrogen), followed by a wash with PBS-T, 15 mM Tris, 0.1× PBS, and 0.05× PBS. Slides were centrifuge dried and then scanned by using a ScanArrayGx (PerkinElmer, Waltham, MA, USA). Images were saved as TIF files.

The fluorescence signal of each spot was digitized with the Scan Array Express Microarray Analysis System (PerkinElmer), exported as comma-delimited files, and quantified as Z-scores^52^ following the following formula:

$${S}_{i}=lo{g}_{2}(\frac{Median(Spot)}{Median(Local\,Background)})$$

$${Z}_{i}=\frac{{S}_{i}-Median({S}_{blank})}{Median\,Absolute\,Deviation({S}_{blank})}$$

$$Median\,Absolute\,Deviation=median(|{Y}_{i}-median({Y}_{i})|),$$

where i is the sample, and in each array spots represent technical replicates for each peptide, and PBS + DMSO spots are the local background.

### Statistical analysis

All data processing, quality control, and analyses were performed in R v3.5.1. Unless otherwise noted, results were considered statistically significant if the two-sided nominal p-value was less than the 0.05. After the MFI was converted to binding scores, batch effect was quantified using PVCA, with the principal component threshold set to 0.8^49^.

#### Adjusting for plate effect

Under the assumption that samples are randomly allocated to plates, the binding for epitope j and sample i (Y_ij_ with j = 1, …, k; i = 1, …, n) can be written as:

$${{\rm{Y}}}_{{\rm{ij}}}\sim 1+{{\rm{Z}}}_{{\rm{j}}}{{\rm{\gamma }}}_{{\rm{j}}}+{{\rm{X}}}_{{\rm{ji}}}{{\rm{\beta }}}_{{\rm{j}}}+{{\rm{\varepsilon }}}_{{\rm{ij}}}$$

were Z is a n_x_P-1 matrix where each column p (2, …, P) is Z_i_ = [1 if sample i is in plate p; 0 otherwise], β is a P-1 dimensional vector containing the plate effect with respect to the reference plate (Plate1), X is the design matrix representing known experimental factors which effect on binding we wish to study, and the residuals ε_ij_ contain any other unknown sources of variation and random fluctuations. Under this model, random allocation of samples to plates will preclude collinearity between Z and X and the P-1 coefficients associated to the plate effect γ, can be unbiasedly estimated (as γ^*^) using classical linear regression or linear mixed effect models. As such, the binding can be adjusted as:

$${{\rm{Y}}}^{\ast }={\rm{Y}}-{Z{\rm{\gamma }}}^{\ast }$$

In a completely randomized experiment, where triplicates were randomly positioned among the 96 wells, a term Z_w_ω_i_ with ω_i_ a 95-dimensional vector was added in the model. Since no position effect was detected (Fig. 2A,B), we recommend not to include well position in plate adjustments. Of note, in experiments with repeated measures (patients followed over time) we recommend to keep samples from the same patient in the same plate and use our freely available web-based application to design a properly randomized microplate experiment^50^.

For the data presented here, plate effect for each peptide was estimated by fitting mixed-effects linear models with “plate” plus either “visit by treatment” for milk or “visit by diagnosis” interaction terms for peanut, and patient-specific random effect and compound symmetric correlation structure using functions from *limma* package^58,78^.

For the reliability analysis a two-way ICC for agreement was used to assess reliability across triplicates, and an ICC for consistency when assessing reproducibility across the laboratories. According to the ICC and Kappa guidelines^51^, the thresholds were defined as excellent (0.75–1.00), good (0.60–0.74), fair (0.40–0.59), and poor (<0.40). Unsupervised clustering of the samples was performed using spearman correlation as a distance metric and “average” agglomeration algorithm.

For the BBEA versus MIA comparisons sample reliability across days was evaluated with the two-way ICC for agreement. Epitope detection was carried out by comparing the differences (𝛿) in binding between positive and negative pool using a moderated paired t-test. Effect size was estimated as 𝛿/SD(𝛿) and epitopes were considered detected if p < 0.05, with no adjustment for multiple hypotheses testing, given the small sample size. As a sensitivity analysis, the same comparisons were made after adjusting BBEA and MIA data for batch effect, i.e. experimental date.

To evaluate the relationship between epitope-specific antibody responses and milk OFC dose, we focused on seven milk IgE-specific epitopes with Pearson correlation less than −0.50 with the OFC dose. The association between the average binding of the 7 peptides for either IgE or IgG4 and the OFC dose (<2 g, 3–10 g, 10 g of milk protein) was evaluated using an ordinal logistic regression. A classification tree with 4 terminal nodes was constructed with the *tree*^79^ R package using IgE and IgG4 as predictors of the binary OFC outcome (10 g versus <10 g).

IgE, IgG4, and IgG4-IgE binding to all 50 peanut epitopes was modeled for peanut allergic and non-allergic patients using linear models in the *limma* framework, and epitopes with FCH≥1.5 and FDR <0.05 were considered statistically significant. To be consistent with the literature, the IgG4-IgE is referred to as a “ratio”; however, since the binding was quantified in the logarithmic scale, the difference between the two antibodies rather than their quotient was calculated.

## Supplementary information

Supplementary Figures and Tables

## Data Availability

The datasets generated during and/or analyzed during the current study are available from the corresponding author on reasonable request.
