# Author Correction: Novel Bead-Based Epitope Assay is a sensitive and reliable tool for profiling epitope-specific antibody repertoire in food allergy

**DOI:** 10.1038/s41598-020-58951-2

**Published:** 2020-02-13

**Authors:** Maria Suprun, Robert Getts, Rohit Raghunathan, Galina Grishina, Marc Witmer, Gustavo Gimenez, Hugh A. Sampson, Mayte Suárez-Fariñas

**Affiliations:** 1 Department of Pediatrics, Allergy and Immunology, Icahn School of Medicine at Mount Sinai, New York, NY USA; 2 Department of Population Health Science and Policy, Icahn School of Medicine at Mount Sinai, New York, NY USA; 3 AllerGenis LLC, Hatfield, PA USA; 4 Department of Genetics and Genomic Sciences, Icahn School of Medicine at Mount Sinai, New York, NY USA

Correction to: *Scientific Reports* 10.1038/s41598-019-54868-7, published online 05 December 2019

The Acknowledgements section in this Article is incomplete.

“We would like to thank Jill Gregory for the illustration of the BBEA assay. We also thank the clinical teams of the milk OIT trial and CoFAR2 study: Robert Wood, MD, Jennifer Kim, MD, Robert Lindblad, MD, Kari Nadeau, MD/PhD, Alice Henning, MSc, Peter Dawson, PhD, Marshall Plaut, MD, Scott H. Sicherer, MD, Tamara Perry, MD, Stacie Jones, MD, Donald Leung, MD/PhD, and Wesley Burks, MD - for their collaboration on data generation, data management and expertise. The study (H.S. and M.SF.) was funded in part by a grant from National Institute of Allergy and Infectious Diseases (NIAID, AI-66738), the David H. and Julia Koch Research Program in Food Allergy Therapeutics, and AllerGenis LLC. AllerGenis was allowed to read and comment on the manuscript, but had no role in study design, data collection and analysis, decision to publish, or preparation of the manuscript. Other funding agencies had no role in study design, data collection and analysis, decision to publish, or preparation of the manuscript.”

should read:

“We would like to thank Jill Gregory for the illustration of the BBEA assay. We also thank the clinical teams of the milk OIT trial and CoFAR2 study: Robert Wood, MD, Jennifer Kim, MD, Robert Lindblad, MD, Kari Nadeau, MD/PhD, Alice Henning, MSc, Peter Dawson, PhD, Marshall Plaut, MD, Scott H. Sicherer, MD, Tamara Perry, MD, Stacie Jones, MD, Donald Leung, MD/PhD, and Wesley Burks, MD - for their collaboration on data generation, data management and expertise. The study (H.S. and M.SF.) was funded in part by a grant from National Institute of Allergy and Infectious Diseases (NIAID, AI-66738), the David H. and Julia Koch Research Program in Food Allergy Therapeutics, and AllerGenis LLC. M.S. was funded by the Integrated Pharmacological Sciences Training Program grant from the National Institute of General Medical Sciences (NIGMS, T32GM062754). AllerGenis was allowed to read and comment on the manuscript, but had no role in study design, data collection and analysis, decision to publish, or preparation of the manuscript. Other funding agencies had no role in study design, data collection and analysis, decision to publish, or preparation of the manuscript.”

