# Supplementary material for: Novel Bead-Based Epitope Assay is a sensitive and reliable tool for profiling epitope-specific antibody repertoire in food allergy
Source: Sci Rep. 2019 Dec 5;9:18425. doi: 10.1038/s41598-019-54868-7 (PMC6895130; doi:10.1038/s41598-019-54868-7)
Supplement: Supplementary file 1 — Supplementary Figures and Tables [file 41598_2019_54868_MOESM1_ESM.pdf]

Supplementary Material

Novel Bead-Based Epitope Assay is a sensitive and reliable tool for profiling epitope-specific antibody repertoire in food allergy

Maria Suprun[1,2], Robert Getts[3], Rohit Raghunathan[2], Galina Grishina[1], Marc Witmer[3], Gustavo Gimenez[1], Hugh A. Sampson[1***], Mayte Suárez-Fariñas[2,4***]

1. Department of Pediatrics, Allergy and Immunology, Icahn School of Medicine at Mount Sinai, New Yok, NY, USA
2. Department of Population Health Science and Policy, Icahn School of Medicine at Mount Sinai, New Yok, NY, USA
3. AllerGenis LLC, Hatfield, PA, USA
4. Department of Genetics and Genomic Sciences, Icahn School of Medicine at Mount Sinai, New Yok, NY, USA

Supplementary Figure S1. Plate effect of the IgG4 data. **A, B.** On the left, PCA and PVCA plots for the 4 milk and 28 peanut IgG4 plates, showing that 2% and 31% (quantified as the weighted average proportion variance, WAPV) of the variability in the data is attributable to the plate effect. PCA plots on the right show that after the adjustment, this effect is eliminated and estimated for both milk and peanut experiments. On the PCA plots points represent individual samples done in triplicates colored by the plates (batches).

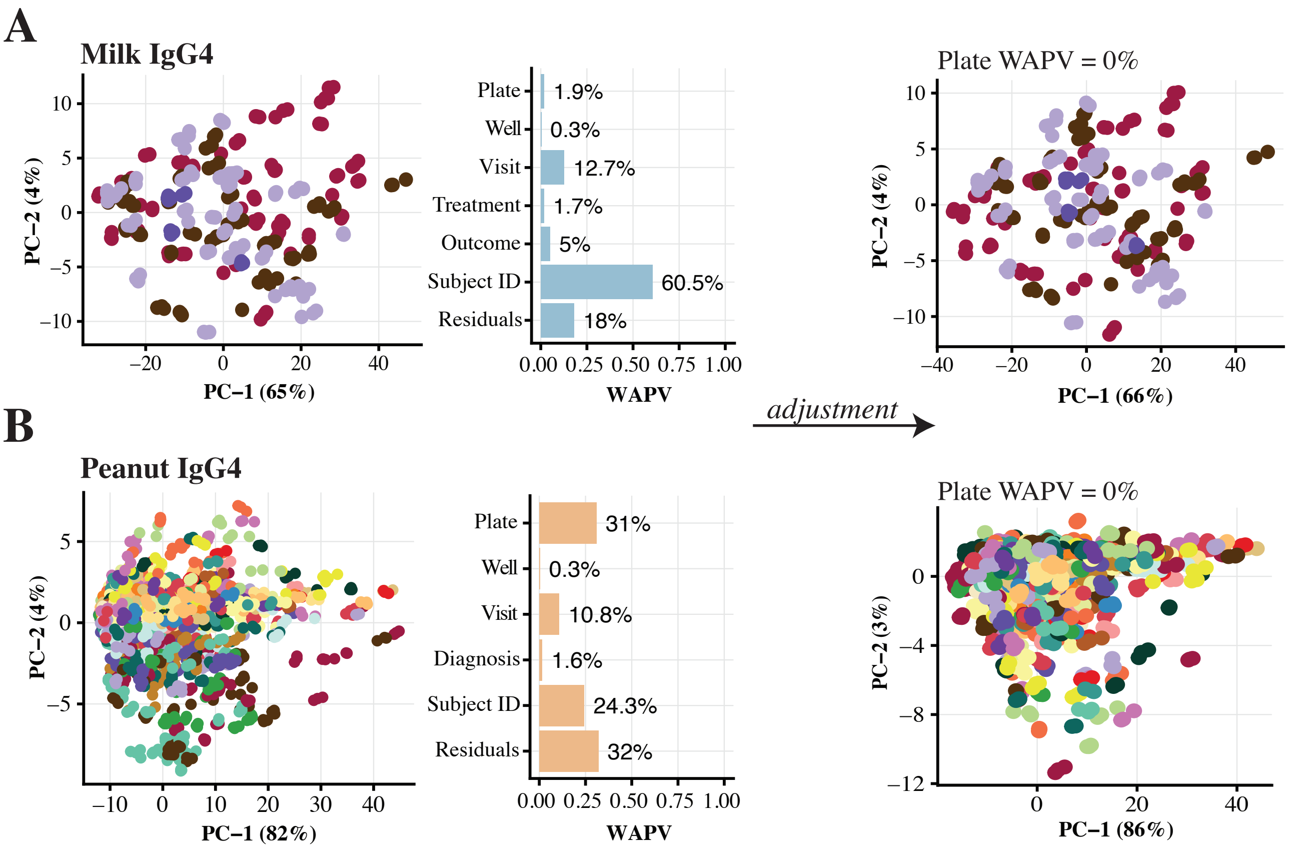

Supplementary Figure S2. Estimation of the plate effect for every epitope of IgE and IgG4 peanut experiments. **A, B.** Heatmaps representing plate coefficients estimated by fitting linear mixed-effects models (a coefficient of zero is indicative of no effect) show that the effect of the plate is not the same across epitopes. **C, D.** Scatter plots of the averaged plate coefficients (plate effect) and binding standard deviation for every epitope. Colors represent mean binding, with higher values colored in red. These plots show that epitopes that have larger plate effect are the ones with the larger standard deviations (variability), that in turn are more common for epitopes with the higher average binding.

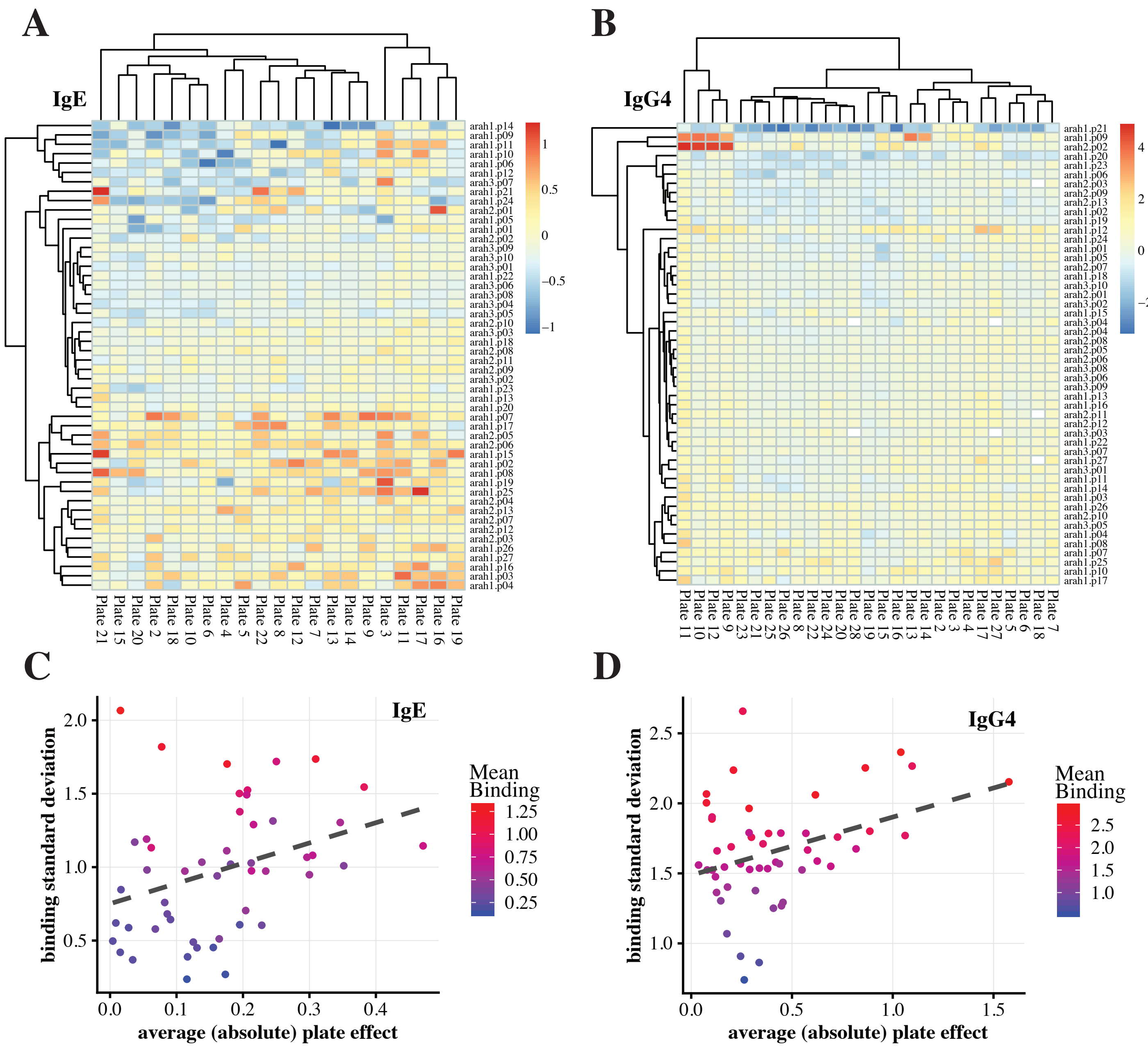

Supplementary Figure S3. Reliability and reproducibility of the IgG4 binding to peanut epitopes. **A.** PCA plot of the technical triplicates colored by the sample type. **B.** Phylogenetic tree of the technical triplicates constructed with the Spearman correlation as distance metric and “average” agglomeration algorithm. **C.** Average agreement ICC with 95% CI across triplicates for all samples within three centers. These results indicate high reliability of the BBEA with triplicates from the same samples clustering together and having high agreement. **D.** PVCA plot shows that the center contributes to a small proportion of total variance (WAPV=29.8%), with the majority of variance explained by the sample type (WAPV=67%). **E.** Average consistency ICC with 95% CI across the 3 centers for all 8 samples. **F.** Average consistency ICC with 95% CI across the 3 centers for all 50 epitopes. **G.** Phylogenetic tree of the technical triplicates after the data were adjusted for the batch effect; constructed with the Spearman correlation as distance metric and “average” agglomeration algorithm. These results show that BBEA has high reproducibility across independent laboratories.

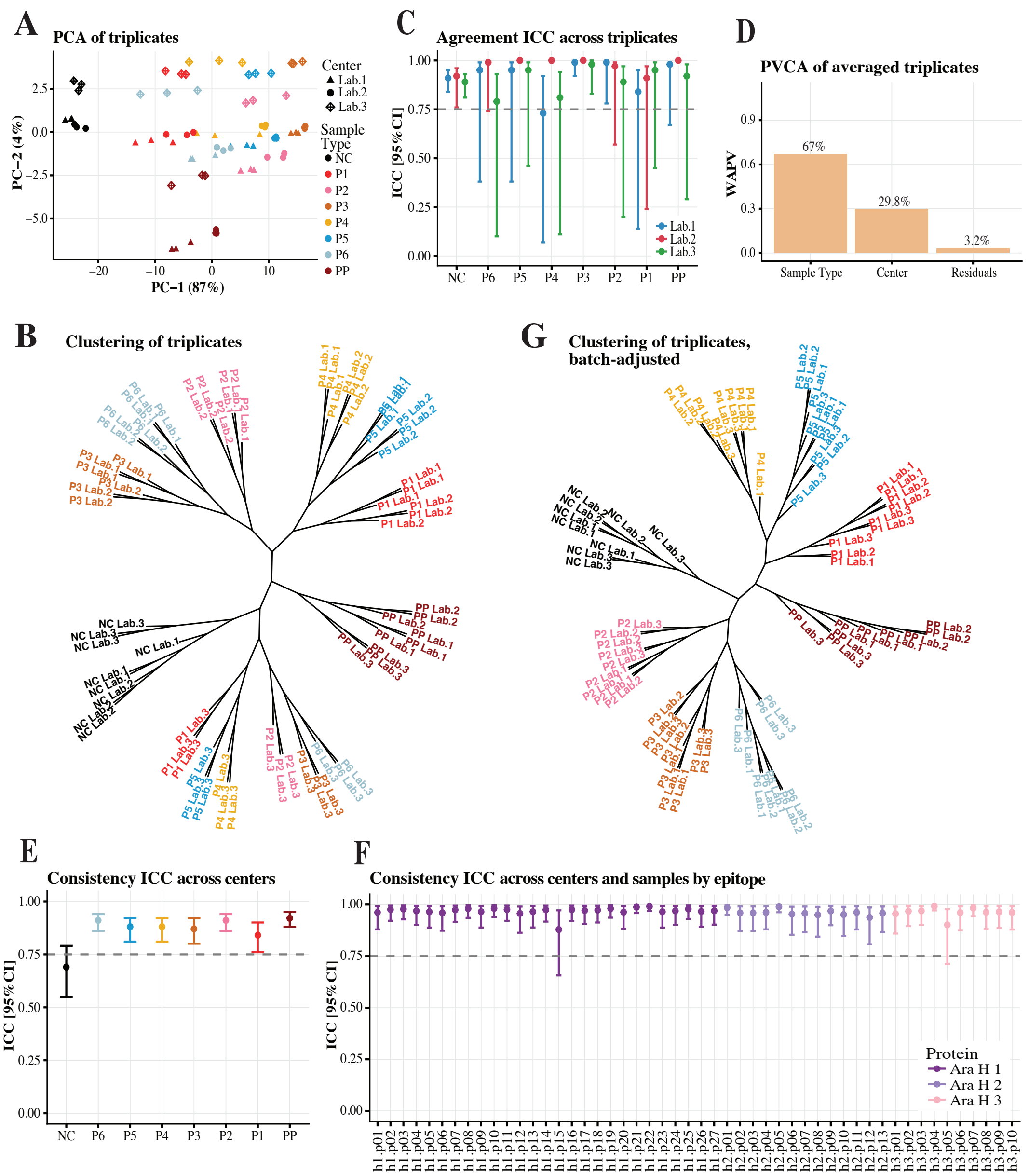

Supplementary Figure S4. Benchmarking of BBEA against MIA using milk epitope-specific IgE for batch adjusted data. **A.** Spearman correlation between replicates with higher color intensity representing stronger correlation (red - positive, blue - negative; all correlations were significant, p<.05). On average, correlation between replicates is close to 1.0 for the BBEA and around 0.7 for MIA. **B.** Average agreement ICC with 95% CI across the two days is higher for BBEA (orange color) compared to MIA (grey color). **C.** Boxplot of the effect sizes shows that BBEA can detect smaller difference. **D.** Scatter plot of the effect sizes show that BBEA detected 66/66 milk epitopes compared to 14/66 detected by MIA. Epitope detection threshold was set to p<.05.

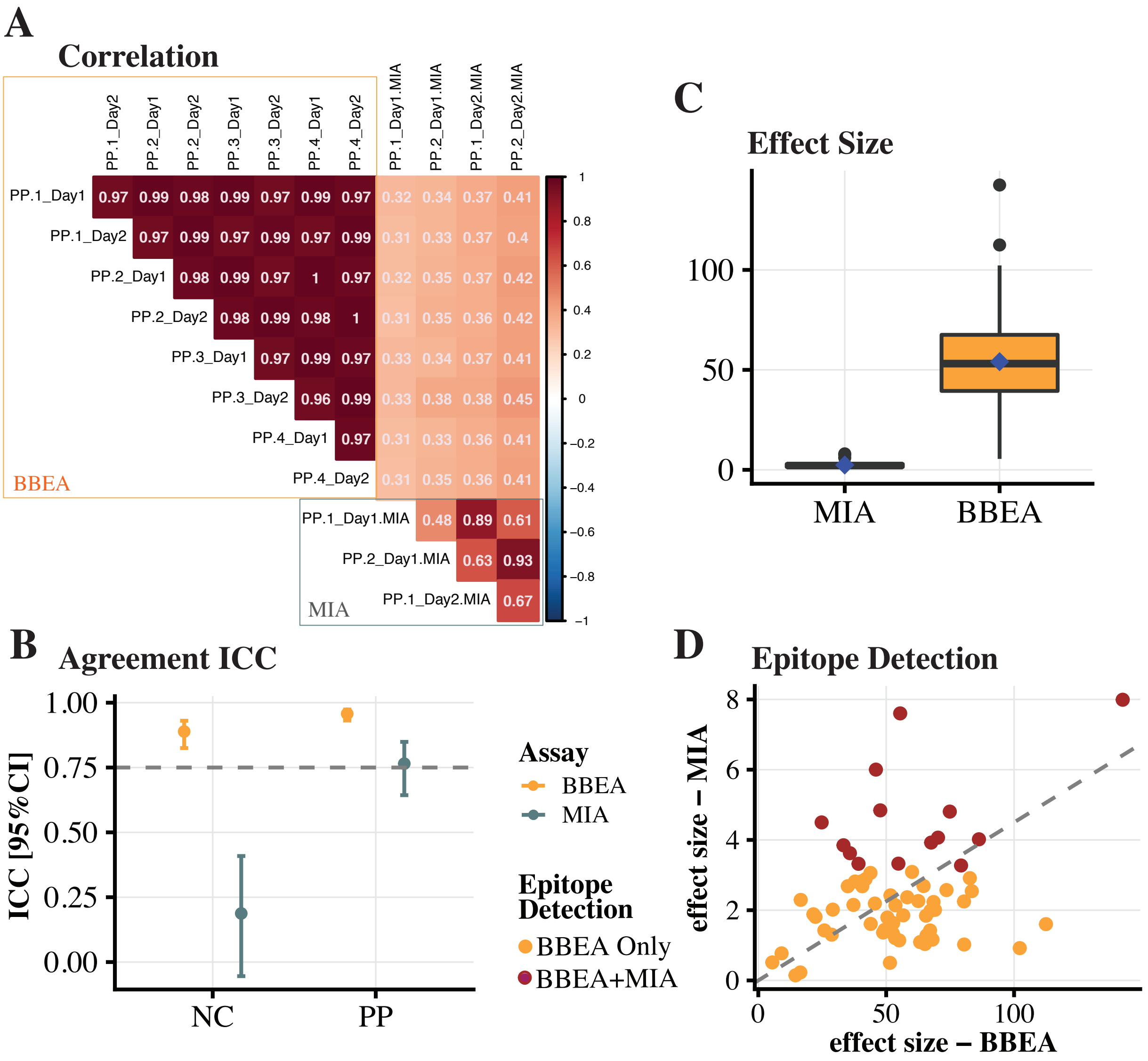

Supplementary Table S1. Reliability metrics for epitope-specific IgG4. The agreement among IgG4 (S3C Fig) replicates is more variable compared to the IgE result (Fig 3C), when evaluated through the ICC for agreement, which quantifies variability across the identity line. This variation in agreement ICC is due to a small additive shift in one of the replicates and not to an increase in variability in the binding measurements. The ICC for consistency (which quantifies variation across a shifted identity line Y=a+X+e), and a pairwise spearman correlation presented in this table shows that the replicates were highly correlated, with the minimum lower bound (LCI) for the consistency ICC of 0.85.

IgG4	ICC - agreement			ICC - consistency			Spearman Correlation		
	ICC	LCI	UCI	ICC	LCI	UCI	replicate1vs2	replicate1vs3	replicate2vs3
NC_Lab.1	0.91	0.84	0.95	0.93	0.89	0.96	0.79	0.84	0.93
NC_Lab.2	0.92	0.76	0.96	0.95	0.93	0.97	0.91	0.94	0.92
NC_Lab.3	0.89	0.81	0.93	0.90	0.85	0.94	0.92	0.94	0.87
P1_Lab.1	0.84	0.14	0.95	0.99	0.99	1.00	1.00	0.99	0.99
P1_Lab.2	0.91	0.24	0.97	0.99	0.99	1.00	1.00	0.99	0.99
P1_Lab.3	0.95	0.45	0.99	1.00	0.99	1.00	0.99	1.00	0.99
P2_Lab.1	0.99	0.78	1.00	1.00	1.00	1.00	1.00	0.99	1.00
P2_Lab.2	0.97	0.57	0.99	1.00	1.00	1.00	0.99	0.99	1.00
P2_Lab.3	0.89	0.20	0.97	0.99	0.99	1.00	0.99	0.99	1.00
P3_Lab.1	0.99	0.92	1.00	1.00	1.00	1.00	1.00	1.00	1.00
P3_Lab.2	1.00	1.00	1.00	1.00	1.00	1.00	1.00	1.00	0.99
P3_Lab.3	0.98	0.83	1.00	1.00	1.00	1.00	1.00	1.00	0.99
P4_Lab.1	0.73	0.07	0.92	1.00	0.99	1.00	1.00	0.99	0.99
P4_Lab.2	1.00	0.99	1.00	1.00	1.00	1.00	1.00	0.99	1.00
P4_Lab.3	0.81	0.11	0.94	0.99	0.99	1.00	0.99	1.00	1.00
P5_Lab.1	0.95	0.38	0.99	1.00	1.00	1.00	0.99	1.00	1.00
P5_Lab.2	1.00	1.00	1.00	1.00	1.00	1.00	1.00	1.00	1.00
P5_Lab.3	0.95	0.46	0.99	1.00	0.99	1.00	0.99	1.00	1.00
P6_Lab.1	0.95	0.38	0.99	1.00	1.00	1.00	1.00	1.00	1.00
P6_Lab.2	0.99	0.74	1.00	1.00	1.00	1.00	1.00	1.00	1.00
P6_Lab.3	0.79	0.10	0.93	0.99	0.98	0.99	0.99	0.99	0.99
PP_Lab.1	0.98	0.67	0.99	1.00	1.00	1.00	1.00	1.00	0.99
PP_Lab.2	1.00	1.00	1.00	1.00	1.00	1.00	0.99	0.99	1.00
PP_Lab.3	0.92	0.29	0.98	0.99	0.99	1.00	0.99	0.99	0.99
